# Closing the loop – the role of pathologists in digital and computational pathology research

**DOI:** 10.1002/2056-4538.12366

**Published:** 2024-03-10

**Authors:** Tilman T Rau, William Cross, Ricardo R Lastra, Regina C‐L Lo, Andres Matoso, C Simon Herrington

## Abstract

An increasing number of manuscripts related to digital and computational pathology are being submitted to *The Journal of Pathology: Clinical Research* as part of the continuous evolution from digital imaging and algorithm‐based digital pathology to computational pathology and artificial intelligence. However, despite these technological advances, tissue analysis still relies heavily on pathologists' annotations. There are three crucial elements to the pathologist's role during annotation tasks: granularity, time constraints, and responsibility for the interpretation of computational results. Granularity involves detailed annotations, including case level, regional, and cellular features; and integration of attributions from different sources. Time constraints due to pathologist shortages have led to the development of techniques to expedite annotation tasks from cell‐level attributions up to so‐called unsupervised learning. The impact of pathologists may seem diminished, but their role is crucial in providing ground truth and connecting pathological knowledge generation with computational advancements. Measures to display results back to pathologists and reflections about correctly applied diagnostic criteria are mandatory to maintain fidelity during human–machine interactions. Collaboration and iterative processes, such as human‐in‐the‐loop machine learning are key for continuous improvement, ensuring the pathologist's involvement in evaluating computational results and closing the loop for clinical applicability. The journal is interested particularly in the clinical diagnostic application of computational pathology and invites submissions that address the issues raised in this editorial.

Digital and computational pathology‐based manuscripts are increasingly being submitted to *The Journal of Pathology: Clinical Research* [1, 2, 3, 4, 5]. This underpins the impact of this technology from basic science to clinically relevant diagnostic application [6, 7, 8] – the scope of our journal. Recent advances in image digitization have simplified collaboration between pathologists for research purposes, for example interobserver studies and consensus in diagnostically challenging cases. Picture archiving and communication systems have been shown to be equivalent to conventional microscopy techniques, with only minimal limitations, including micrometer z‐stacks, polarization and focus points, among others, that require additional research. After this prerequisite for a digital workflow, the arrival of morphometry‐based algorithms from various companies and open‐source systems have advanced more precise evaluation of biomarkers. In particular, immunohistochemistry can be analyzed more precisely as counts per area or cell numbers compared with traditional cumbersome and time‐consuming enumerations or quick eyeball estimates. However, known interpretive elements like threshold setting, hotspot selection, and segregation of tissue classes have remained or even become more important with these quantitative approaches. Emerging novel histogram‐based representations have helped to overcome debated scoring systems like immune reactive score or *H*‐score. Lastly, computational and artificial intelligence models have been developed within an interdisciplinary framework of contributing pathologists, data scientists, engineers, and informaticians [6]. The system ‘learns deeply’ how to obtain the best results for a given training set based on dynamically and iteratively optimized multifactorial zero‐one decisions that are obscured to the trainer. Deep learning is now an integral part of the scientific repertoire to study tissues, but is strongly dependent on one factor: the trainer.

The input of pathologists in terms of annotations frequently serves as a starting point for intensified data analysis. In reflecting about this important but frequently underestimated task during the evaluation of submitted articles, three important elements can be highlighted: granularity, time issues, and responsibility for the interpretation of computational results.

Granularity refers to the detail in which annotations are made by a pathologist. In a frequently cited review article in this journal from the PathLake Group, four layers of annotation are mentioned [9], namely case level, regional annotations, cellular compositions, and attributions from elsewhere (e.g. synoptic report elements, molecular pathology, etc.). Frequently under‐appreciated, whole slide image (WSI) selection already represents a pathologist annotation as not all information about a case is present on a single slide. Additionally, subcellular information such as nucleoli, mucin depletion, mitoses, nuclear grooves, and pleomorphism are of interest. These layers of granularity parallel the general pathology knowledge of searching for features at certain magnifications under the microscope. It is important to bring this knowledge of diagnostic criteria together with the applied computational technique; for example, as a rule of thumb, tile size definition should match the diagnostic criteria, for example architectural criteria might not be resolved at high magnification (small tile size) and vice versa. Of note, attribution of diagnostic classes to noninformative tiles, such as ductal carcinoma *in situ* attribution without technically visible myoepithelium, should prompt our scientific curiosity. Technical solutions like random tile cropping, tile shifting, and analysis at different scanning resolutions in parallel could optimize the correct tile size creation.

Looking at time as a factor, time constraints due to shortage of pathologists come to the fore. Several techniques have been developed to reduce input time from pathologists during annotation tasks [10]. Representative tissue classes determined from a few cells can be upscaled with locally run algorithms to whole slides based on morphological similarities. In computational methods, pathologists are asked to sort tile stocks to one class and decide upon resulting borderline cases with few iterations. The aggregation of such tile stocks could be provided by a convolutional network agnostic to any attribution. This so‐called unsupervised method allows calculations to run on the images hypothesis‐free [11]. However, these methods frequently take advantage of former hidden annotations by pathologists, including slide or tissue microarray core selection, molecular data from selected tissue areas, or the prior histopathology report containing prognostic information related to the image. Given these hidden annotations, the term unsupervised could even be questioned; the term weakly supervised should be considered to acknowledge the human input. Finally, in telling the items apart, the expert pathologist performs diagnostics in its original Greek sense. Hence, the role of the pathologist might be dual in providing a gold standard with annotations, but also in interpreting the output of the computational methods. Full annotations at the cellular level are the most valuable reference but are hard to obtain. The dedicated time for slide annotations seems to correlate indirectly with the experience of the annotator in research practice; this task may be performed by residents or students rather than by field experts in pathology or board certified pathologists.

Regarding responsibility for the interpretation of computational results, the impact of pathologists seems to be reduced in the literature as technological aspects are emphasized more strongly. A histopathological baseline is postulated, which is – as pathologists know – always preliminary, and observer‐ and task‐dependent. Marking the tumor is performed differently to obtain molecular pathology data, quantify epithelial biomarkers, or study stromal elements. The so‐called ground truth can be set with descending reproducibility and stability from normal human anatomy to pathology, disease prognostication, and ultimately therapeutic prediction. Of note, conversely, oncological interest increases with these steps, but is more likely to vary over time. Therefore, conventional pathology research is still needed to improve diagnostic criteria, test robustness with interobserver reproducibility studies, and seek consensus. The rapid evolution of pathological knowledge is evident by the rhythm of World Health Organisation (WHO) classifications, which currently have a turnover time of approximately 6 years [12]. Emerging entities, novel ancillary tools, molecular definitions, and shifts in biomarker thresholds are only some elements that continuously refine the pathological gold standard, leading to so‐called diagnostic shifts in healthcare. Access to unprocessed image data according to FAIR (findability, accessibility, interoperability and reusability) principles [13, 14] is key and will ensure future adaptations of algorithms in terms of data monitoring. This demand is strongly supported by the SPIRIT‐Path guidelines, which outline the contribution of pathology to clinical trials [15, 16, 17].

Strengthening the connection between pathological knowledge generation and the computational field is crucial. In the literature, the lack of communication between pathologists and other scientists can be seen in various examples, including relevant diagnostic classes being missed in the computational training setup (known as hidden stratification), assembly of large case series that fail to represent emerging entity subtypes reinforcing outdated practices, and computational methods not being tested for relevant mimickers and differential diagnoses, to mention a few [18, 19]. Pathologists should commit themselves to the AI‐driven transition and regain their central role as tissue experts and evaluators of digital and computational methods. Conversely, pathologists should accept that their descriptive and sometimes vague language forms the basis for future machine reading and will undergo embedding in ontologies and structured reproducible elements, such as the International Collaboration on Cancer Reporting [20].

In their current form, computational methods are reproductive in nature though at intended higher precision. This applies to well‐known contests such as the CAMELYON trial for lymph node metastasis in breast cancer [21] or the PANDA challenge for Gleason grading of prostate cancer [22]. However, progress beyond the pathological *status quo* will only be achieved if pathological and computational research seeks to surpass the boundaries of current knowledge. The most suitable approach for pathology is human‐in‐the‐loop machine learning, which is the opposite of streamlining applications away from pathologists once annotations are made.

Closing the loop to clinical applicability brings in the pathologist once again during the evaluation of computational methods. The terms explainability, causability, and interoperability were recently discussed in this journal in an invited review [23]. There are many innovative ways to display computational results, for example UMAP (uniform manifold approximation and projection), vector graphics, and cell distribution heatmaps, which should be discussed with pathologists and interpreted with their input. Additionally, interactions with the computational output could initiate an additional iteration to compare deep features with corrected handcrafted features in an effort to test their reliability and robustness. Furthermore, the interdisciplinary research team may generate mathematical representations of histopathological features (e.g. morphometric parameters) that can be compared quantitatively with the computational result, unlocking the black box of AI and allowing conventional criteria to be weighed and sorted in a better way. We argue that loops and iterations are key for continuous improvement practices and strongly believe that ensuring that the pathologist remains in the loop makes the difference.

## Call for papers

The involvement of pathologists is central to evaluation of the clinical utility of digital and computational pathology methods. *The Journal of Pathology: Clinical Research* invites the submission of original articles using digital and computational approaches for relevant clinical applications. We are interested in papers that focus on clinical application in any field of pathology, but would give priority to submissions that:

1. Precisely describe the testing and validation cohorts used according to REMARK guidelines [24].
2. Critically evaluate and describe annotations made by pathologists, from case and block selection to cell‐level annotations
3. Match WHO entity‐specific essential and desirable criteria with the applied computational approach.
4. Delineate the limitations of the computational algorithm with the inclusion of known differential diagnoses, mimickers and pitfalls as a separate challenging case series.
5. Close the loop with final evaluation of computational versus conventional pathological features.

We look forward to receiving your manuscript through the journal submission system: https://mc.manuscriptcentral.com/jpathclinres.

## Author contributions statement

All authors contributed to the writing of this editorial.
